# A Disintegrin and Metalloprotease (ADAM): Historical Overview of Their Functions

**DOI:** 10.3390/toxins8040122

**Published:** 2016-04-23

**Authors:** Nives Giebeler, Paola Zigrino

**Affiliations:** Department of Dermatology and Venerology, University of Cologne, Cologne 50937, Germany; nives.giebeler@uk-koeln.de

**Keywords:** ADAM, disintegrin, SVMP

## Abstract

Since the discovery of the first disintegrin protein from snake venom and the following identification of a mammalian membrane-anchored metalloprotease-disintegrin implicated in fertilization, almost three decades of studies have identified additional members of these families and several biochemical mechanisms regulating their expression and activity in the cell. Most importantly, new *in vivo* functions have been recognized for these proteins including cell partitioning during development, modulation of inflammatory reactions, and development of cancers. In this review, we will overview the a disintegrin and metalloprotease (ADAM) family of proteases highlighting some of the major research achievements in the analysis of ADAMs’ function that have underscored the importance of these proteins in physiological and pathological processes over the years.

## 1. Some Generalities

**Figure 1:**
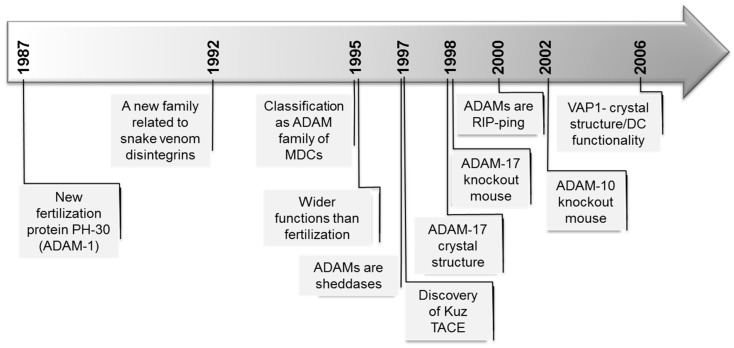
Timeline of key events in ADAMs research. The dates correspond to the publication year of the first article related to the event.

ADAMs (a disintegrin and metalloproteinases), originally also known as MDC proteins (metalloproteinase/disintegrin/cysteine-rich), belong to the Metzincins superfamily of metalloproteases. The timeline of key events in ADAM research is shown in Figure 1.

**Figure 2:**
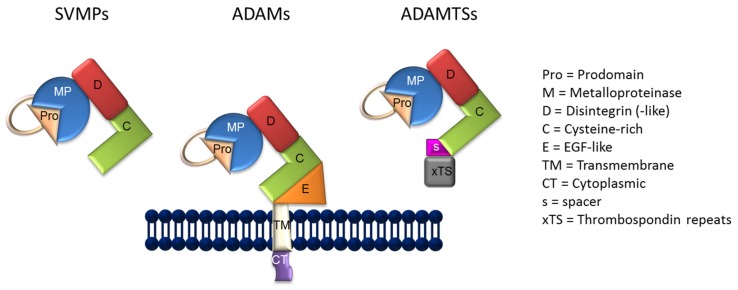
General structure of ADAMs (a disintegrin and metalloproteinases), ADAMTSs (a disintegrin and metalloproteinases with thrombospondin motif), and SVMPs (snake venom metalloproteases).

Together with snake venom metalloproteases (SVMPs), ADAM and ADAMTS (a disintegrin and metalloproteinases with thrombospondin motif) proteins form the M12 (MEROP database; https://merops.sanger.ac.uk/) Adamalysin subfamily of metallopeptidases. ADAMs have been detected in various species, from *Ciona intestinalis* to mice and humans [1]. Phylogenetic and molecular evolutions studies on these proteins have identified several gene duplications followed by pseudogene formation and/or positive selection of those genes mostly related to reproduction thus ensuring survival of the species [2]. Duplications and speciation have probably contributed to the divergence of SVMP and ADAM from the common ancestor gene [3]. Similarities in domain organization and sequences exist between the ADAMs and the P-III SVMPs [4]. Both protein families contain a pro-domain, a metalloproteinase and a disintegrin domain, and a cysteine domain. The latter in ADAMs has cell adhesive and fusogenic potential. ADAMs also contain an EGF-like repeat, a transmembrane domain, and a cytoplasmic tail (Figure 2). In addition, the tails of some ADAMs have intrinsic signaling activity and regulate proteolysis [5]. Alternative splicing of ADAMs produces proteins with different localization and activity [6].

**Figure 3:**
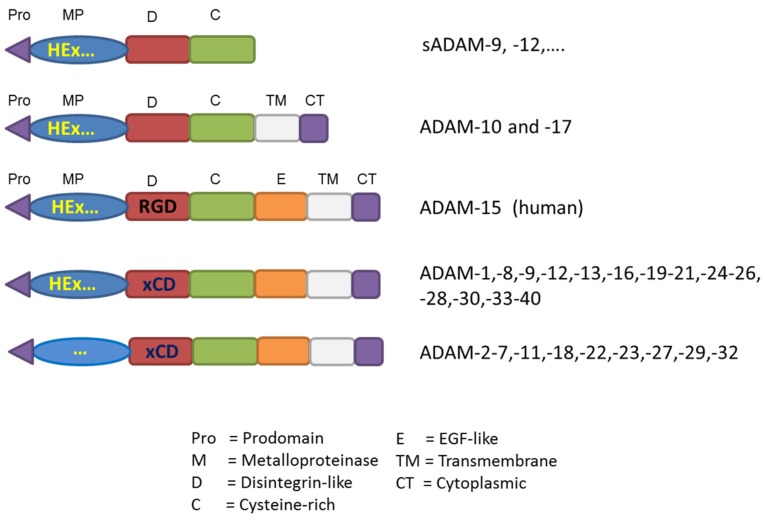
Protein domain structure comparison between ADAMs. Metalloproteinase domains with consensus sequence HEx (HExGHxxGxxHD, HEx…) are predicted to be proteolytic active (“…”, lack of a consensus sequence). Only human ADAM-15 contains a RGD amino acid sequence, all other ADAMs contain a conserved consensus binding motif xCD in their disintegrin-like domains. Soluble ADAMs, sADAM.

The label “disintegrin” was initially given to describe snake venom cysteine-rich, RGD-containing proteins able to adhere to integrins and inhibit platelet aggregation and cause hemorrhage in snake bite victims [7]. Similar to SVMPs, ADAMs adhere to integrins even though their binding sequence mostly contains an aspartic acid-containing sequence ECD (or xCD sequence) instead of the typical RGD amino acid sequence, except for human ADAM-15 (Figure 3). For this reason these domains are referred to as “disintegrin-like” domains [8]. Structural analysis of resolved structures of the ADAM and SVMP domains has been extensively reviewed elsewhere [9,10]. Only half of the known ADAMs contain a catalytic-Zn binding signature for metalloproteases (HExGHxxGxxHD) in their metalloprotease domain and can potentially be catalytically active. Those mammalian ADAMs that are catalytically active use a cysteine-switch mechanism to maintain enzyme latency [11]. Interestingly, the pro-domain not only is implicated in this process, but is also important for the correct protein folding and intracellular transport through the secretory pathway as shown for example for ADAM-9, -12, and -17 [12,13,14].

## 2. ADAMs Functions

### 2.1. ADAMs and Cell Adhesion

The initial studies on ADAMs focused on the function of the disintegrin domain in cell-cell and cell-matrix interactions. As for the SVMP, the disintegrin-like domain of ADAMs was also supposed to bind to integrins on the cell surface. Similarly to SVMP, human, but not mouse ADAM-15 contains an integrin binding motif RGD in the disintegrin domain. However, human ADAM-15 can also bind integrin in a RGD-independent manner as shown for the binding of α9β1 integrin [15]. An extensive review on ADAM-15 structural and functional characteristics has been provided by Lu and colleagues [16]. Several *in vitro* studies using recombinant domains, mutation studies, or peptide sequences supported this role for a variety of ADAMs and suggested their function as cellular counter receptors [17,18]. This ADAMs-integrin interaction was not receptor-specific as each ADAM could interact with several integrins and may depend on the cell type.

Additional studies suggested that interactions with substrates and integrin receptors are not mediated solely by the disintegrin domain, but additional sequences outside this domain are involved in binding. In this respect, Takeda and colleagues analyzed the crystal structure of VAP1 (vascular apoptosis-inducing protein-1, a P-III SVMP), which has a conserved MDC structure, and identified the disintegrin-loop of this protein packed inside the C-shaped MDC architecture, which is therefore not available for binding [19]. Interestingly, these authors identified another sequence, the highly variable region (HVR) of the cysteine rich domain as a potential protein-protein adhesive interface [19]. It is therefore possible that the cysteine-rich domain drives adhesion or complements the binding capacity of the disintegrin domain possibly conferring specificity to the mediated interactions. The disintegrin and the cysteine-rich domains can also mediate cellular interactions via binding not only to integrins, but also to heparansulfate proteoglycans, such as syndecans [20]. Despite all these studies, the question remained whether the ADAM–integrin interactions identified *in vitro* could be relevant *in vivo*.

Few studies analyzed the crystal structure of ADAM proteases (reviewed in [9,10]). An example is the analysis of the crystal structure of the mature ADAM-22 ectodomain, one of the catalytically inactive ADAMs. In this analysis, ADAM-22 displays a four-leafed clover structure of four domains, the MDCE (metalloproteinase/disintegrin-like/cysteine-rich/EGF-like), without the pro-domain. According to this, the authors proposed that ADAMs function is modulated by dynamic structural changes in M domain and DCE domains that allow for the opening and closing of the protein configuration [21]. Electron microscopy studies on the full-length ADAM-12-s showed a similar four-leafed structure. In this structure, as for ADAM-22, the pro-domain is an integral domain of mature ADAM12 non-covalently associated to M domain [22].

Analysis of the crystal structure of ADAM-17 metalloproteinase domain bound to a hydroxamic acid inhibitor highlighted some unique features of the active site distinct from matrix metalloproteinases that may contribute to substrate specificity of TACE (TNF alpha converting enzyme) [23].

However, the substrate specificity depends on the entire MDC structure. Guan and colleagues [24] recently performed crystallographic analysis of two new SVMPs: atragin and kaouthiagin-like (K-like). This study pointed out that the MDC structure of atragin is C-shaped whereas that of K-like has an I-shaped structure depending on the disulfide bond patterns present in the D domain of both enzymes. Thus, the D domain would be important in orientating the M and C domains for their function and substrate specificity [24].

The physiological relevance of the interaction of ADAMs with integrins was clearly shown for the fertilization process. The first studies were done on ADAM-1, previously known as PH-30 (found on guinea pig sperm surface) and later as fertilin alpha (found in mouse and monkey), and on ADAM-2, or fertilin beta [25,26,27]. Interestingly, both proteins lose their metalloproteinase domain during the maturation process and are present on the cell surface exposing directly their disintegrin domain [28]. Initially, the disintegrin-like domain of ADAM-2 was shown to bind to the integrin α6β1 on the egg plasma membrane, and that this binding was required for membrane fusion [29]. Additional ADAMs were suggested to be involved in the egg-sperm membrane fusion fertilization process based on the fact that the first ADAMs discovered (ADAM 1–6) were found to be expressed in mammalian male reproductive organs such as testis and epididymis (reviewed by Cho [30]). However, although the first discovered members of the ADAM family, ADAM-1, ADAM-2, and -3 are critical in mediating fertilization processes, this mediation does not depend on their adhesive and fusogenic potential [31]. Indeed, deficiency of either ADAM-1a or -2 revealed an indirect involvement of these ADAMs in fertilization. Both ADAM-1 and -2 deficient sperms were defective in migration, but the process that leads to cell-cell adhesion and fusion was only minorly altered [32,33,34]. ADAM-2 is not dispensable for the sperm-egg fusion process, however, ~50% decreased fusion sperm to oocytes was detected in the deficient mice [32,35]. Nine years later Yamaguchi and colleagues demonstrated that the ADAM-2–ADAM-3 complex is critical for *in vivo* sperm migration from the uterus to the oviduct [36]. Interestingly, ADAM-1 and -3 are pseudogenes in humans leaving ADAM-2 as the key ADAM mediating human fertilization [37]. Apart from the proposed role in fertilization, ADAMs have been implicated in cell fusion during development as described for ADAM-12, promoting myoblast fusion during myogenesis [38,39].

In addition to mediating interactions with cell surface receptors for adhesive and fusogenic purposes, the disintegrin and cysteine domains were suggested to regulate the proteolytic function of ADAMs as shown for the shedding of interleukin-1 receptor-II by ADAM-17 [19]. Recently, Dusterhoft *et al.* [40] demonstrated that an MDC adjacent to the conserved region in ADAM-17, named CANDIS (Conserved ADAM seventeeN Dynamic Interaction Sequence), is able to bind membranes, thereby regulating shedding activity. Therefore, further studies are necessary to elucidate the precise function of the ADAM disintegrin-loop for physiological functions of ADAMs.

### 2.2. ADAMs Are Active Proteases

By analogy to the SVMPs, that also have a metalloprotease and disintegrin domain, it was suggested that some ADAMs cleave extracellular matrix components. Indeed, early in 1989 Chantry *et al*. found that brain myelin membrane preparations contain a metalloproteinase activity which degrades myelin basic protein (MBP) [41]. The responsible metalloprotease was cloned in 1996 and named MADM/ADAM-10 [42]. In 1994, a metalloprotease implicated in TNFα (tumor necrosis factor alpha) processing was described [43] and identified a few years later as a member of the Adamalysin family, ADAM-17. ADAM-17 was and described simultaneously by two research groups as the enzyme that releases membrane bound tumor necrosis factor (TNF)-α precursor to a soluble form earning a name TACE (TNF alpha converting enzyme) [44,45]. ADAM-17 had a unique structure with very similar sequence to ADAM-10 [44,46].

In the following years, new discoveries on the proteolytic functions of ADAM-10 and -17 opened a larger field of research activities. ADAM-10 was identified in Drosophila, called Kuzbanian (gene *Kuz*), and is required for the cleavage of Notch and its ligand Delta leading to lateral inhibition and to neuronal fate specification during neural development [47,48,49,50]. Given its highly conserved structure in Drosophila, Xenopus, and C. Elegans, ADAM-10 was proposed to be of great importance in vertebrate development. ADAM-10 has a wide variety of functions including ECM degradation, localized shedding of various cell surface proteins, and influence on cell signaling patterns (reviewed in [51]). Importantly, shedding activities mediated by ADAM proteases may affect both cell autocrine and paracrine signaling. Indeed, shedding may occur on the surface of one cell or two adjacent cells, and the outcome may not only affect the receptor but also the ligand-bearing cell, thus resulting in the generation of reciprocal signals. As a consequence of cell surface protein shedding, the released soluble ectodomain with signaling or decoying function may act on another distant cell (reviewed in [52]).

#### 2.2.1. ADAMs in EGFR Transactivation

Over time ADAMs were shown to process a wide variety of substrates among which are the EGFR (epidermal growth factor receptor) ligands. Aberrant EGFR activation has been frequently found in hyper proliferative diseases such as cancer, and the discovery of the ligand-dependent EGFR signal transactivation pathway may explain how autocrine signaling loops involving GPCR (G-protein coupled receptor) ligands are likely to contribute to and drive autocrine EGFR stimulatory mechanisms [53]. Prenzel and colleagues [54] first showed that EGFR transactivation by ligand involved the activity of a metalloproteinase. The transactivation involved an upstream signal acting through a G-protein-coupled receptor that activated a metalloprotease to shed an EGF receptor ligand. The result of this activity ultimately led to the modulation of mitogen responses [54]. The first studies directly implicating ADAM-10 in EGFR transactivation were presented by two groups in 2002 [55,56]. Several other studies have followed and identified additional ADAMs that fulfil this function in various cell types and tissues including ADAM-12, -15, and -17 [57]. ADAMs have been implicated in the shedding of six out of the seven known EGFR ligands (transforming growth factor (TGF)α, EGF, HB-EGF, betacellulin, epiregulin, and amphiregulin) [52]. ADAM activation by GPCRs, and the mechanisms and pathophysiological role of ADAM-dependent EGFR transactivation have been reviewed elsewhere [57,58].

#### 2.2.2. RIPping by ADAMs

Upon shedding of the extracellular domain of some cell surface proteins, the activity of a second protease cleaving within the membrane leads to the release of an intracellular fragment of the protein, generally the cytoplasmic tail, which exerts signaling activity. This process is known as Regulated Intramembrane Proteolysis (RIP). RIP starts with the release of the protein ectodomain by the activity of ADAMs that is followed by intramembrane-cleavage and final release of the intracellular stub within the cell. Aspartyl proteases, S2P-metalloproteases, and rhomboid serine proteases catalyze the intramembrane cleavage [59]. RIPping is an evolutionary highly conserved mechanism to release messenger peptides from transmembrane proteins [60]. The classical examples for RIP activity are the processing of amyloid-precursor-protein (APP) and Notch signaling [20]. Ligation of Notch to its ligand leads to a conformational change exposing the protease-sensitive sequence to ADAM-10 that sheds and releases Notch ectodomain. This primes truncated Notch for additional intramembrane cleavage by gamma-secretase, thereby releasing the intracellular domain (NICD, notch intracellular domain) that translocates to the nucleus and mediates transcriptional activities (reviewed by [61]). ADAM-10 and -17 can cleave the extracellular domain of Notch when it is bound to ligands like Delta or Jagged, and from this follows subsequent intramembrane processing of Notch by γ-secretase activity [62,63]. Recently, Notch was shown to enhance its own activity by transcriptionally inducing expression of furin, which in turns leads to the activation of MMP (matrix metalloproteinase)-14 and ADAM-10 and ultimately to the amplification of Notch signaling [64]. This would further provide a mechanism of signal amplification during tumor progression.

APP also undergoes RIPping, most likely in a ligand-independent fashion. Among ADAMs, ADAM-9, -10, and -17, were shown to cleave APP *in vitro* acting as α-secretases [65]. Processing of the APP extracellular domain by either α- or β-secretase is followed by intramembrane proteolysis by γ-secretases, but, in the case of the APP, the intracellular role of the intracellular released fragment remains unclear [59]. However, whereas fragments generated by α-secretases are physiological, those released by β-secretase are neurotoxic and key factors in Alzheimer’s disease [66].

RIPping is not limited to Notch and APP, as this mechanism is active for several other proteins shed by ADAMs. ADAM-10 is able to mediate RIPping of a variety of additional proteins such as Notch2, Notch3, N-, and E-cadherin, and ADAM-17 may mediate RIPping of EpCAM and ErbB4 [67,68,69,70,71]. Interestingly, there is also evidence for a reciprocal RIPping of ADAMs themselves. For example, RIPping of ADAM-10 may be executed by ADAM-9 and -15 leading, upon presenilin intramembrane proteolysis, to the release of the ADAM-10 intracellular domain (ICD) that localizes to nuclear speckles [72]. ADAM cleavage sites are usually in close proximity to *O*-glycosylation sites. Most recently Goth and colleagues investigated the potential of site-specific *O*-glycosylation on peptides from known ADAM-17 substrates and proposed that *O*-glycosylation might co-regulate ectodomain shedding by ADAMs [73].

#### 2.2.3. Inhibition of Proteolytic Activity

Apart from being active towards matrix metalloproteinase, TIMP (tissue inhibitors of metalloproteinases) were shown to be active towards ADAMs [74]. After the first discovery of ADAM-17 inhibition by TIMP-3 [75], additional ADAMs were shown to be selectively inhibited by TIMPs. For example, ADAM-10 can be inhibited by TIMP-1 and -3 but not by TIMP-2 and -4, and ADAM-8 and -9 are not sensitive to inhibition by TIMPs at all [20]. ADAM-9, -10, and -17 prodomains can inhibit protease activity once released from the activated enzyme [76,77,78]. A number of synthetic inhibitors binding to the catalytic zinc ion, but with a broad inhibitory spectrum towards ADAMs and MMPs, have been described. Although the majority of those inhibitors could inhibit both MMPs and ADAMs, some were relatively selective for specific ADAMs. An example is the INCB3619 which inhibits ADAM-8, -9, -10, -17, and -33, but with lower IC50 for ADAM-10 and -17 [79]. Other synthetic inhibitors include CGS 27023, GW280264, and GI254023 displaying a certain degree of specificity for ADAM-9, -10, and -17 [14,80]. An interesting recent report indicated that glycosylation of substrates may also play a role in modulating ADAM activity, thus glycosylation of TNF-α enhanced ADAM-8 and -17 activities and decreased ADAM-10 activity [81]. The importance of this study was the discovery of a novel class of ADAM-17-selective inhibitors that act via a non-zinc-binding mechanism. Thus, additional type of inhibitory molecules targeted to these unique exosites within ADAM structures could be used for specific protease targeting [81].

## 3. Lessons from *in Vivo* Models

An important milestone in ADAM research was the generation and analysis of *in vivo* models for ADAM deficiency. Particularly, the generation of ADAM-10 and -17 *in vivo* mutants helped by elucidating their substrate specificity in a physiologically relevant context. ADAM-10 knockout mice die at day 9.5 of embryogenesis due to multiple defects of the developing central nervous system, somites, cardiovascular system, and missing Notch signaling thus providing a key evidence for the functional role of ADAM-10 in Notch processing *in vivo* [82]. ADAM-17 knockout mice die between embryonic day 17.5 and the first day of birth, not due to a lack of TNF-α shedding or dysregulated TNF-α signaling, but because of a lack in processing of multiple EGFR-ligands including TGF-α, HB-EGF, and amphiregulin. These *in vivo* findings are extensively reviewed elsewhere [52,83,84]. Recently the two new proteins iRhoms 1 and 2 (inactive Rhomboid proteins, catalytically inactive members of the rhomboid family of intramembrane serine proteases) were identified as upstream ADAM-17 regulators, controlling the substrate selectivity of ADAM-17-dependent shedding [85,86]. Additionally, truncated iRhom 1 or iRhom 2 enhance ADAM-17 activity towards TNFR shedding, thereby triggering resistance against TNF-induced cell death [87]. In agreement with these functions, the knockout mouse of both iRhoms closely resembles ADAM-17-deficient mouse phenotype, with lack of functional ADAM-17 as well as lack of EGFR phosphorylation [88].

Although ADAM-10 and -17 have become the most prominent members of this protease family, there are a few other ADAMs with significant importance in mouse development *in vivo*. ADAM-22 deficiency leads to early death due to severe ataxia and hypomyelination of peripheral nerves indicating an important role of this protease in the development of the peripheral nervous system [89]. In addition, ADAM-19 is important for heart development as 80% of ADAM-19 deficient animals die postnatally from congenital heart defects [90]. Deletion of ADAM-12 results in 30% neonatal lethality and a 30% reduction in brown adipose tissue [91], and ADAM-11 knockout animals show deficits in spatial learning, motor coordination, and altered nociception responses [92,93]. Unexpectedly, ADAM-9-deficient mice show no obvious developmental phenotype [94], but in adulthood, 20 months after birth, mice display retinal degeneration [95]. However, depletion of ADAM-9 showed its implication in pathological induced retinal vascularization, in skin repair, and in melanoma growth [96,97,98]. Similarly, ADAM-15 depletion showed reduced pathological neovascularization and tumor metastasis [99,100].

Given the early lethality of ADAM-10 and -17, the generation of conditional tissue specific mutants has permitted the opportunity to learn more about the role of these proteases in tissue homeostasis. For instance, nestin-Cre driven ADAM-10 deletion, with inactivation of ADAM-10 in neuronal progenitor cells, has identified ADAM-10 as the main α-secretase for APP [101]. Further, ADAM-10 depletion in epidermis leads to disturbed epidermal homeostasis [102] whereas its depletion in the endothelial cells leads to various vascular defects in organs conducible to defects in endothelial cell fate determination [103]. Overall, ADAM-10 has emerged as one of most important members of the ADAM family with broad spectrum of protein shedding (for further studies see [104,105]).

Specific loss of ADAM-17 function in epidermis leads to pronounced defects in epidermal barrier integrity and development of chronic dermatitis, closely resembling mice with epidermal loss of the EGFR [106]. Furthermore, ADAM-17 ablation in the endothelial cell progeny does not appear to be required for normal developmental angiogenesis or vascular homeostasis, but for pathological neovascularization [107]. Lisi and colleagues have recently summarized the phenotypes of ADAM-17 genetic models [108].

Thus, more studies with tissue-specific genetic targeting of ADAMs or *in vivo* challenging will be necessary to clarify protease or adhesive function of these proteins in an *in vivo* physiological and pathological context.

## 4. Impact on Human Disease

In various human cancers there is an increased expression of ADAM-8, -9, -10, -15, -17, -19, and -28 [109,110].

ADAM-9 null mutations were found in patients from families with recessively inherited cone-rod dystrophy (CRD), an inherited progressive retinal dystrophy. Interestingly, retinal degeneration in adulthood was also detected in the ADAM-9-deficient mouse [95].

ADAM-17 is involved in human inflammatory diseases such as rheumatoid arthritis (a systemic inflammatory autoimmune disorder), the Guillain-Barré syndrome (an acute autoimmune polyneuropathy), multiple sclerosis (an inflammatory, autoimmune, demyelinating disease of the central nervous system), and systemic lupus erythematosus (an autoimmune disease affecting nearly all organs) [111]. ADAM-17 deficiency is very rare in humans, as it has been reported only in three patients, and causes severe multiorgan disorders [112,113,114].

ADAM-33 has been identified as the susceptibility gene for asthma and airway hyper responsiveness, but its biological function is yet unclear [115].

In Alzheimer’s disease ADAM-10 could have beneficial properties, as APP processing by ADAM-10 reduces both APP cleavage by BACE1 and β-amyloid generation, a physiologically active APP fragment clumping into neurotoxic aggregates. Indeed, accumulation of β-amyloid peptide in the brain leads to development of Alzheimer’s disease [116]. β-amyloid peptide is produced by the processing of APP by β-secretases, but a soluble form of APPα is produced by the action of ADAM-9, -10, and -17 which oppose the adverse effects of the β-amyloid peptide [117,118,119]. Thus, the upregulation of various zinc metalloproteinase activities may represent a possible alternative therapeutic strategy for the treatment of the disease [120].

## 5. Future Perspectives

ADAMs are implicated in a variety of cellular functions *in vitro* and *in vivo*, and many substrates for some of these proteases are already known. However, additional substrates are yet to be discovered, and further protein degradation analyses may help to identify the protease implicated. This analysis, together with the analysis of processing characteristics, such as shedding followed by intramembrane proteolysis and generation of an ICD signaling fragment, will further identify downstream effects of such proteolytic events.

Over the years it has become clear that ADAMs are implicated in several diseases, and because of the broad specificity of several synthetic inhibitors to MMPs or ADAMs targeting the metalloproteinase activity, it is now important to develop alternative strategies for *in vivo* targeting. Recombinant pro-domains or the generation of specific antibodies may help with increasing targeting specificity. Moreover, targeting exosites has recently gained importance in drug development. One of the outstanding questions that still need to be addressed is, what are the protein modifications on substrates or substrate structure that determine specificity of binding and enzyme efficacy? Protein modifications have just started to be analyzed, and will certainly lead to new clues for selective targeting strategies.
